# Buffalo Medical and Surgical Association

**Published:** 1885-10

**Authors:** 


### Society Reports.

### Buffalo Medical and Surgical Association.

*Meeting of September 1, 1885.*

The President, Dr. C. G. Stockton, in the chair; Dr. F. R. Campbell, Secretary. Present—Drs. Bartlett, Hopkins, Van Peyma, Frederick, Wm. Ring and W. W. Potter.

There being no paper for discussion, the President called for voluntary communications.

Dr. C. C. Frederick reported the case of a negress who, about ten months ago, received a kick in the vulva. Inflammation and swelling, followed by a purulent discharge from the vagina, resulted from this injury. The pain and swelling soon disappeared, but the discharge continued, and incontinence of urine supervened, which has continued to the present time. Dr. Frederick found, on examination, that almost the entire vesico-vaginal septum had sloughed away, but as there was plenty of tissue remaining in the vagina, he considered it feasible to attempt a cure by operation.

Dr. W. W. Potter agreed with Dr. Frederick in considering it possible to restore the vesico-vaginal wall in this case.

Cholera infantum, dysentery and whooping cough were reported as the prevailing diseases. It was the opinion of several of the gentlemen present that the general health of the city was better than at the corresponding period last year.

